# Peanut Stunt Virus and Its Satellite RNA Trigger Changes in Phosphorylation in *N. benthamiana* Infected Plants at the Early Stage of the Infection

**DOI:** 10.3390/ijms19103223

**Published:** 2018-10-18

**Authors:** Barbara Wrzesińska, Lam Dai Vu, Kris Gevaert, Ive De Smet, Aleksandra Obrępalska-Stęplowska

**Affiliations:** 1 Institute of Plant Protection—National Research Institute, Department of Entomology, Animal Pests and Biotechnology, Władysława Węgorka 20, 60-318 Poznań, Poland; b.wrzesinska@iorpib.poznan.pl; 2 Department of Plant Biotechnology and Bioinformatics, Ghent University, Technologiepark 927, 9052 Ghent, Belgium; lamvu@psb.vib-ugent.be (L.D.V.); ive.desmet@psb.vib-ugent.be (I.D.S.); 3 VIB Center for Plant Systems Biology, Technologiepark 927, 9052 Ghent, Belgium; 4 Department of Biomolecular Medicine, Ghent University, B-9000 Ghent, Belgium; kris.gevaert@vib-ugent.be; 5 VIB Center for Medical Biotechnology, B-9000 Ghent, Belgium

**Keywords:** signaling, virus infection, *Nicotiana benthamiana*, *Peanut stunt virus*, plant-virus interactions, satellite RNA, phosphoproteome, phosphoproteomics, RNA turnover, phosphorylation

## Abstract

Signaling in host plants is an integral part of a successful infection by pathogenic RNA viruses. Therefore, identifying early signaling events in host plants that play an important role in establishing the infection process will help our understanding of the disease process. In this context, phosphorylation constitutes one of the most important post-translational protein modifications, regulating many cellular signaling processes. In this study, we aimed to identify the processes affected by infection with *Peanut stunt virus* (PSV) and its satellite RNA (satRNA) in *Nicotiana benthamiana* at the early stage of pathogenesis. To achieve this, we performed proteome and phosphoproteome analyses on plants treated with PSV and its satRNA. The analysis of the number of differentially phosphorylated proteins showed strong down-regulation in phosphorylation in virus-treated plants (without satRNA). Moreover, proteome analysis revealed more down-regulated proteins in PSV and satRNA-treated plants, which indicated a complex dependence between proteins and their modifications. Apart from changes in photosynthesis and carbon metabolism, which are usually observed in virus-infected plants, alterations in proteins involved in RNA synthesis, transport, and turnover were observed. As a whole, this is the first community (phospho)proteome resource upon infection of *N. benthamiana* with a cucumovirus and its satRNA and this resource constitutes a valuable data set for future studies.

## 1. Introduction

Viruses are among the most important causal agents of infectious diseases in animals and plants. Plant viruses are composed of a protein coat and a nucleic acid core [1]. Their life cycle is strictly associated with a host cell environment and uses the host’s biochemical machinery. Therefore, viruses need hosts in order to multiply and spread, and thus they are considered as parasites [1]. Viruses, by invading susceptible plant host cells, lead to a pathogenesis process, which results in the disturbance of the host physiology, causing disease symptoms. The interactions between viral and plant factors lead to engagement of many host proteins in defense responses against infectious agents [2,3,4].

*Peanut stunt virus* (PSV) is a plant virus that belongs to the worldwide present *Cucumovirus* genus (*Bromoviridae* family). PSV infects many legume plants, including peanut (*Arachis hypogaea* L.), soybean (*Glycine max* (L.) Merr.), or bean (*Phaseolus vulgaris* L.), as well as Solanaceae species, including the model plant *Nicotiana benthamiana*. PSV is a positive sense, single stranded RNA ((+)ssRNA) virus and its genome consists of three genomic and two subgenomic strands. Additionally, certain PSV strains contain a noncoding satellite RNA particle (satRNA) [5]. RNA1 and RNA2 encode components of the viral replicase complex 1a and 2a, respectively. Moreover, RNA2 has an additional open reading frame for the 2b protein, synthesized from the subgenomic RNA4A, which is known to participate in viral movement and silencing suppression [6,7]. RNA3 is dicistronic and encodes a movement protein (3a) and a coat protein (CP). The latter is synthesized from subgenomic RNA4 [8].

PSV-P, a well-studied PSV strain belonging to the I subgroup of PSVs [5], is associated with a satRNA, a 393 nt (+)ssRNA molecule that depends on its helper virus for replication. SatRNAs are divided into two groups: large satRNAs encoding a single nonstructural protein and smaller ones (as satRNA of PSV) that do not encode functional proteins [9]. SatRNAs are able to modulate helper virus titer (usually decrease) and symptom severity (usually attenuate), which depends on the strains of the helper virus, the sequence of the satRNA, the host [9,10], and environmental conditions [11]. The satRNA can influence the plant metabolism, resulting in significant changes in protein levels, including those of proteins important for photosynthetic activities and carbon metabolism [12].

Such observed changes in protein levels gave insight into the molecular mechanisms involved in host–virus–satRNA interactions [12]. Still, however, little is known about early signaling and the post-translational modifications (PTMs) occurring during plant virus-induced pathogenesis [13]. PTMs lead to the induction of protein structural changes, modulation of their activities, changes in subcellular localization, stability, and interactions with other proteins and molecules [14]. Among many PTMs, phosphorylation is one of the major control mechanisms for protein activity in plant–pathogen interactions and the best characterized PTM regulating cellular signaling processes [15,16]. Phosphorylation of serine, threonine, and tyrosine is considered a crucial factor in the integration and relay of signals within plant cells and one of the most abundant covalent modifications of proteins [17]. Reversible protein phosphorylation is a major mechanism of signal transduction that is mediated by protein kinases and protein phosphatases, thereby regulating many biological processes [18]. Moreover, this PTM has been reported to be associated with reactions toward abiotic and biotic factors (e.g., defense responses against pathogens) [19]. Phosphorylation changes in the host proteome under virus infection are investigated more thoroughly in humans or animals than in plants. Studies on signaling processes mediated by phosphorylation in plants encounter difficulties caused by low abundance and the dynamic nature of phosphorylated proteins. However, significant advances in mass spectrometry-based phosphoproteomics, including phosphopeptide enrichment, detection, and quantification, as well as phosphorylation site localization [20], have recently emerged. There are studies concerning phosphorylation as a regulator of many processes during viral infection in plants; however, these are mainly connected with the phosphorylation status of single plant or viral proteins [21,22,23,24,25,26]. Global changes in the plant phosphoproteome during plant virus infection remain poorly studied [13]. Such studies are, however, highly useful to expand the understanding of the mechanisms of plant–virus interactions.

Here, we analyzed the proteome and phosphoproteome of *N. benthamiana* plants infected with PSV-P or with PSV-P together with satRNA at five days post inoculation (dpi). Our analysis revealed strong differences in the numbers of the phosphorylated proteins and their levels isolated from PSV-P and PSV-P and satRNA-infected *N. benthamiana*. Strong down-regulation in phosphorylation in virus-treated plants (without satRNA) was detected, whereas satRNA addition to virus inoculum caused only a slight decrease in overall phosphorylation levels. Moreover, differentially regulated (phospho)proteins were mostly associated with photosynthesis, carbon metabolism, and RNA processing.

## 2. Results

### 2.1. Experimental Set-Up for Proteome and Phosphoproteome Analyses of PSV-P- or PSV-P and satRNA-Infected N. benthamiana Plants

**Figure 1:**
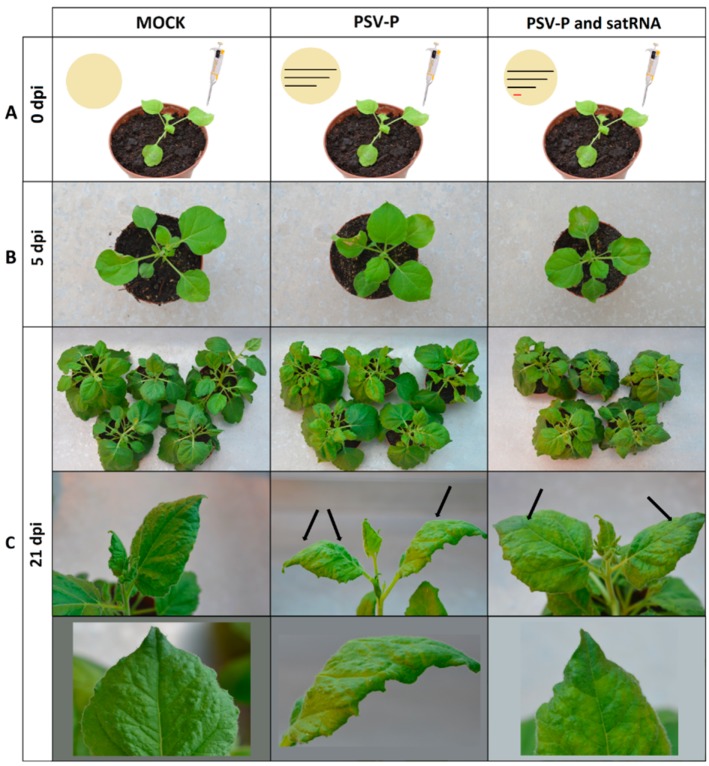
Schematic representation of the infection strategies and resulting phenotypes. (**A**) *N. benthamiana* plants inoculated with inoculation buffer (MOCK), inoculation buffer supplied with in vitro transcripts of peanut stunt virus (PSV)-P genomic strands (black lines) (PSV-P), inoculation buffer supplied with in vitro transcripts of PSV-P genomic strands and satellite RNA (black lines and red line, respectively) (PSV-P and satRNA) (**A**). (**B**,**C**) *N. benthamiana* plants inoculated with the following: inoculation buffer (MOCK), in vitro transcribed PSV-P genomic strands (PSV-P), and in vitro transcribed PSV-P genomic strands and satellite RNA (PSV-P and satRNA) at 5 days post inoculation (dpi) (**B**) and 21 dpi (**C**). Black arrows indicate infection symptoms: chlorosis and leaf malformations generated by PSV-P and chlorosis in PSV-P and satRNA-treated plants (**C**).

To gain insight into the early molecular changes associated with the response of *N. benthamiana* to infection with PSV-P and PSV-P and satRNA, and into the influence of satRNA presence in the virus inoculum, proteome and phosphoproteome analyses were performed. The fifth day post infection (5 dpi), before the rapid rise of the virus accumulation [11], was chosen as a time point for harvesting to reveal early signaling during virus infection. Plants were inoculated with genomic transcripts of PSV-P, PSV-P and satRNA, and mock (inoculated with phosphate buffer–control) (Figure 1A) and harvested at 5 dpi, when the disease symptoms were not yet visible (Figure 1B). After *N. benthamiana* plants were harvested at 5 dpi, PSV-P and satRNA accumulation levels were assessed by means of quantitative reverse transcriptase real-time polymerase chain reaction (RT-qPCR) in order to pick plants for (phospho)proteome analyses. PSV accumulation analysis revealed higher levels or no change of viral RNAs in PSV-P treated plants (Figure S1), which is consistent with previous data on the changes in the proteome of *N. benthamiana* infected with PSV-P with or without satRNA, where a slight decrease in viral RNA accumulation in the presence of satRNA was reported [12]. Plants in which the accumulation level was similar (within the tested condition) were taken for (phospho)proteome analysis.

### 2.2. PSV-P and PSV-P and satRNA Infections Influence the N. benthamiana Proteome

**Figure 2:**
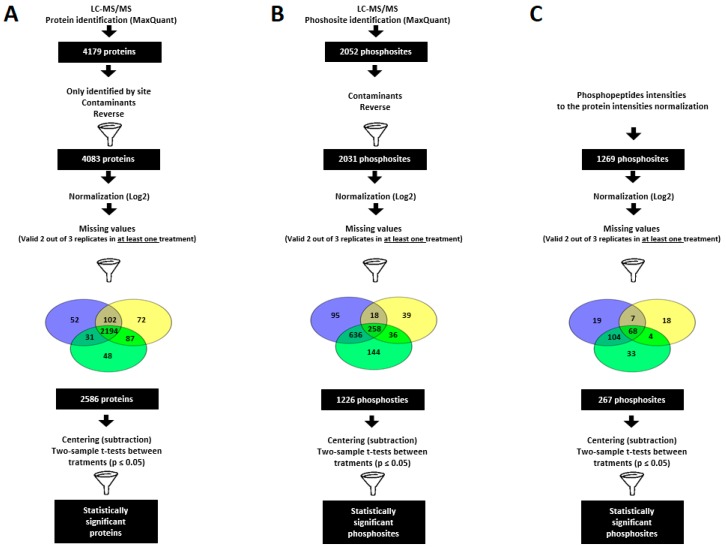
Summary of the data analysis. Data analysis workflow of (**A**) proteome, (**B**) phosphoproteome, and (**C**) normalized phosphoproteome of *N. benthamiana* plants infected with PSV-P and PSV-P and satRNA.

To study the changes in the proteome in response to PSV-P or PSV-P and satRNA, we applied a previously described workflow [27]. In total, 19,830 peptides were identified, which were assigned to 4083 protein groups. Of these, the 2586 proteins present in at least two replicates in at least one condition (Table S1A) were subjected to further analysis, and 93.4% of them were found to be present in all conditions (Figure 2A). To pinpoint differentially abundant proteins between conditions in order to detect proteins referred as PSV-P-, PSV-P and satRNA-, and satRNA-responsive, pairwise two-sample tests (*p* < 0.05) between conditions were performed (Table 1, Table S1C–E). A greater number of PSV-P-responsive proteins (111) was up-regulated, however, addition of satRNA to the virus inoculum caused a strong increase in down-regulated satRNA-responsive proteins number (218).

**Figure 3:**
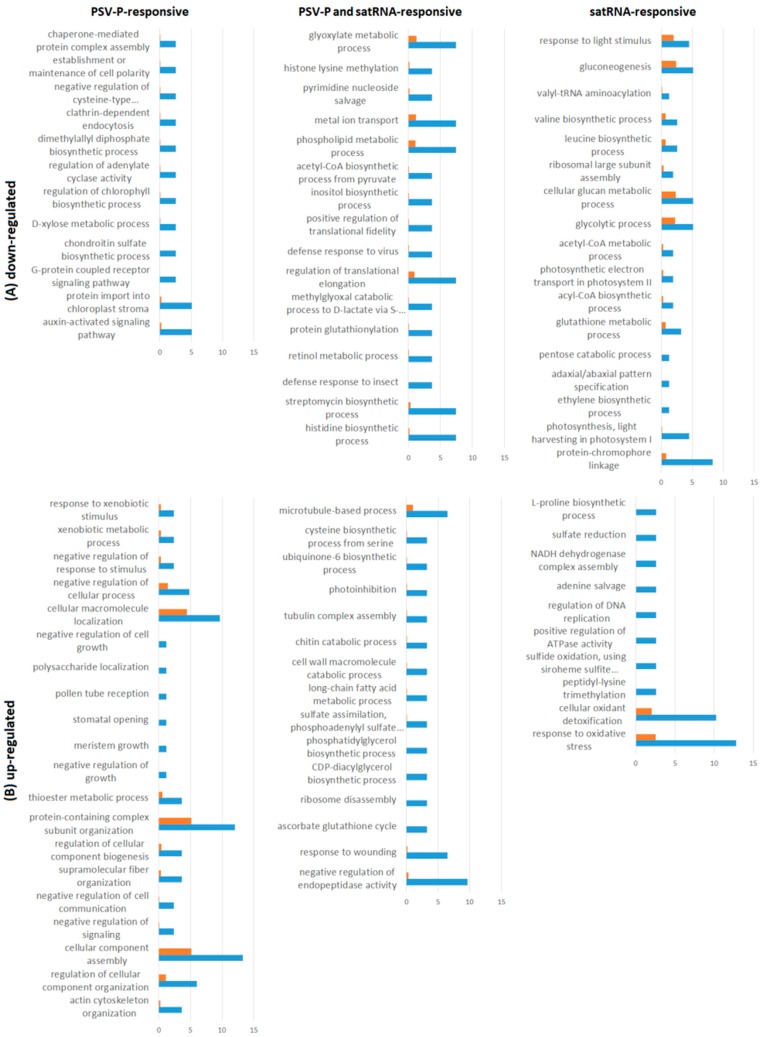
Gene ontology (GO) enrichment for the biological process for proteome analysis. Graphs display GO terms for PSV-P- or PSV-P and satRNA-infected *N. benthamiana* plant proteins. The distribution of GO terms was analyzed separately for down-regulated (**A**) and up-regulated proteins (**B**) using Blast2GO Basic.

To obtain insight into the functions and processes of proteins involved in PSV-P and PSV-P and satRNA infection at the early stage, a gene ontology (GO) enrichment analysis was performed using Blast2GO Basic Software. For this analysis, proteins exclusively present (up- or down-regulated) in one condition (Table S2) were analyzed. The most enriched terms among the down-regulated PSV-P-responsive proteins were auxin-activated and G-protein coupled receptor signaling pathways, protein import into chloroplast stroma, regulation of chlorophyll biosynthetic process, and chaperone-mediated protein complex assembly (Figure 3A). Whereas, the up-regulated terms included actin cytoskeleton organization, negative regulation of signaling, and negative regulation of cell communication (Figure 3B). On the other hand, among the PSV-P and satRNA-responsive proteins, the most enriched GO terms were, in case of down-regulated proteins, histidine biosynthetic process, defense response to virus, and regulation of translation-related terms (regulation of elongation, positive regulation of translational fidelity) (Figure 3A). However, among the up-regulated PSV-P and satRNA-responsive proteins, the most GO enriched terms were negative regulation of endopeptidase activity, ribosome disassembly, cell wall macromolecule catabolic process, tubulin complex assembly, photoinhibition, and microtubule-based process (Figure 3B). Down-regulated satRNA-responsive proteins were enriched in terms associated with photosynthesis (light harvesting in photosystem I, photosynthetic electron transport in photosystem II) and ribosomal large subunit assembly terms (Figure 3A), whereas the enriched terms concerning the up-regulated satRNA-responsive proteins were response to oxidative stress, cellular oxidant detoxification, and positive regulation of ATPase activity (Figure 3B).

**Figure 4:**
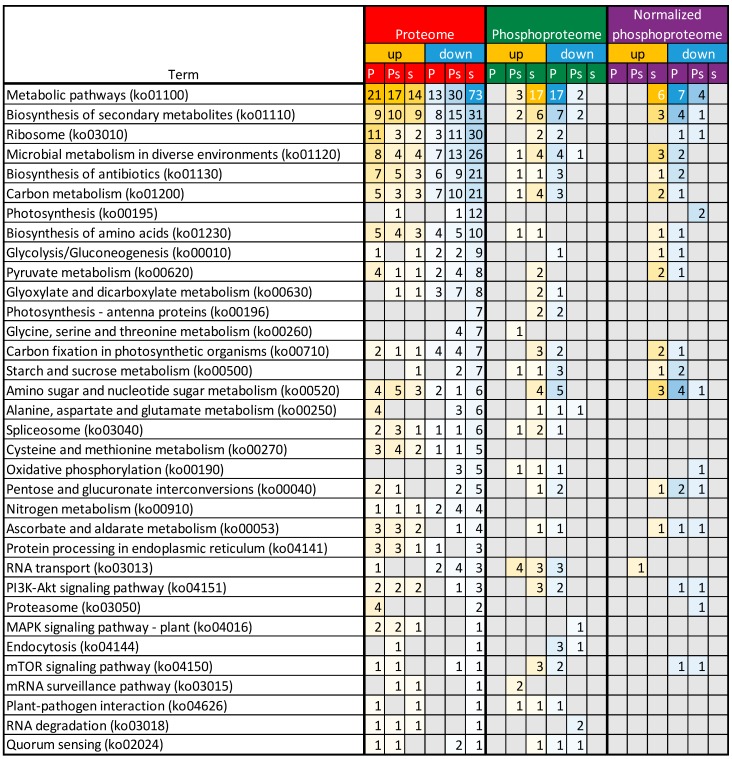
KEGG Automatic Annotation Server (KAAS) analysis. Analysis conducted separately on down- and up-regulated (phospho)proteins during PSV-P and PSV-P and satRNA infection of *N. benthamiana* plants using Blast2GO Pro. P—PSV-P-responsive, Ps—PSV-P and satRNA-responsive, s—satRNA-responsive, MAPK—mitogen-activated protein kinase, TOR—target of rapamycin. Values represent the numbers of repeated terms.

An additional analysis to indicate the affected pathways was conducted using the KEGG (Kyoto Encyclopedia of Genes and Genomes) Automatic Annotation Server (KAAS) tool [28], revealing the most affected pathways by PSV-P, PSV-P and satRNA infection, and satRNA presence in virus inoculum (Figure 4). The highest number of affected pathways concerned down-regulated satRNA-responsive proteins, which correlated with the high number of differentially regulated proteins in this condition. Among the affected pathways, there were ribosome (ko03010); carbon metabolism (ko01200); and photosynthesis-related terms (photosynthesis (ko00195), photosynthesis-antenna proteins (ko00196), and carbon fixation in photosynthetic organisms (ko00710)). The pathway related to ribosomes was strongly down-regulated by satRNA presence in the virus inoculum; however, in PSV-P-treated plants, more proteins related to this this pathway were up-regulated. Additionally, together with ribosome (ko03010), carbon metabolism (ko01200), and biosynthesis of amino acids (ko01230), proteasome (ko03050) was up-regulated in PSV-P-treated plants. Moreover, proteins related to RNA transport (ko03013) were more down-regulated among PSV-P and satRNA-responsive proteins. Signaling pathways such as PI3K–Akt (phosphoinositide 3-kinase–serine/threonine-specific protein kinase) (ko04151), mitogen-activated protein kinase (MAPK) (ko04016), and mTOR (target of rapamycin) (ko04150) were affected to a certain extent by PSV-P or PSV-P and satRNA. Nonetheless, satRNA contributed to down-regulation of few proteins involved in these signaling pathways. Furthermore, pathways related to plant–pathogen interaction (ko04626) were also found.

### 2.3. PSV-P and PSV-P and satRNA Infections Influence the N. benthamiana Phosphoproteome

In total, Ti-IMAC (Ti^4+^-immobilized metal affinity chromatography) enrichment followed by LC-MS/MS (liquid chromatography-mass spectrometry/mass spectrometry) analysis led to the detection of 1373 phosphopeptides containing 2031 phosphosites (Table S1F) located on 1060 phosphoproteins. Surprisingly, the highest number of phosphosites simultaneously detected in two conditions was shared between mock and PSV-P and satRNA samples (636), while the number of phosphosites that overlapped between all three conditions was lower (258) (Figure 2B). To pinpoint proteins with a changed abundance between conditions, two-sample tests (*p* < 0.05) between conditions were performed (Table 1, Table S1H–J). The majority of the PSV-P-responsive proteins (87) was down-regulated in their phosphorylation level, whereas among the satRNA-responsive proteins, a higher number of proteins with up-regulated phosphosites (84) was identified (Table 1).

**Figure 5:**
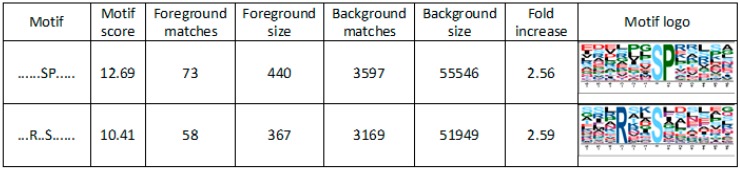
Motif-X analysis performed for overrepresented phosphorylation motifs of significantly regulated phosphosites in PSV-P- and PSV-P and satRNA-infected *N. benthamiana* leaves.

To reveal phosphorylation motifs in significantly regulated phosphosites, Motif-X analysis was conducted. This analysis indicated two potential phosphorylation motifs for phosphoserine: SP and RxxS (Figure 5). The SP motif is a common target for proline-directed kinases, which include mitogen-activated kinases and cyclin-dependent kinases, whereas RxxS is a recognition motif for 14-3-3 proteins [29] that were also found in our data (Table S1F).

**Figure 6:**
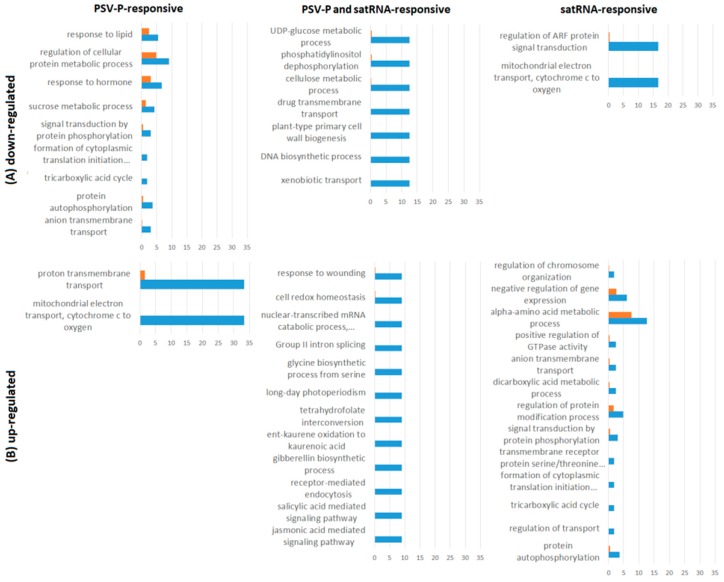
GO enrichment for biological process for phosphoproteins. Data from PSV-P- or PSV-P and satRNA-infected *N. benthamiana* plant phosphoproteins. The distribution of GO terms was analyzed separately for down-regulated (**A**) and up-regulated proteins (**B**) using Blast2GO Basic.

GO enrichment analysis was also performed using the Blast2GO Basic Software. For this analysis, phosphoproteins exclusively present (up- or down-regulated) in one condition (Table S2) were analyzed. This analysis demonstrated that under the biological process category, PSV-P-responsive proteins with down-regulated phosphopeptides were assigned to the formation of the cytoplasmic translation initiation complex, sucrose metabolism, protein autophosphorylation, and signal transduction by protein phosphorylation, while PSV-P-responsive proteins with up-regulated phosphopeptides were enriched in mitochondrial electron transport and proton transmembrane transport terms (Figure 6). Regarding PSV-P and satRNA-responsive proteins with regulated phosphopeptides, the down-regulated ones were enriched in plant-type primary cell wall biogenesis, while among the up-regulated ones, there were jasmonic- and salicylic acid-mediated signaling pathways, nuclear-transcribed mRNA catabolic processes, and nonsense-mediated decay. Additionally, satRNA-responsive proteins with down-regulated phosphopeptides were enriched in mitochondrial electron transport and regulation of ARF (ADP-ribosylation factor) protein signal transduction, whereas the up-regulated terms were enriched in protein autophosphorylation, regulation of transport, formation of cytoplasmic translation initiation complex, transmembrane receptor protein serine/threonine kinase signaling, and signal transduction by protein phosphorylation.

The pathway analysis using KAAS revealed no results for proteins with up-regulated phosphopeptides in PSV-P-treated plants and down-regulated ones in response to satRNA addition to the virus inoculum (Figure 4). The most affected pathways associated with down-regulated PSV-P-responsive and up-regulated satRNA-responsive proteins with regulated phosphopeptides were amino sugar and nucleotide sugar metabolism (ko00520), carbon metabolism (ko01200), RNA transport (ko03013), ribosome (ko03010), and photosynthesis-related pathways (carbon fixation in photosynthetic organisms (ko00710) and antenna proteins (ko00196)). PI3K–Akt (ko04151) and mTOR (ko04150) signaling pathways were altered likewise. Additionally, the up-regulation of phosphopeptides of satRNA-responsive proteins also influenced pyruvate metabolism (ko00620). Moreover, infection with PSV-P and satRNA mostly influenced mRNA surveillance (ko03015) and RNA degradation (ko03018).

### 2.4. A Normalized N. benthamiana Phosphoproteome upon PSV-P and PSV-P and satRNA Infections

In order to pinpoint the significantly deregulated phosphopeptides under virus infection, phosphopeptide intensities were normalized to the protein intensities [27]. This is necessary as changes in phosphopeptide abundance may result from true phosphorylation changes (kinase or phosphatase activity) or changes in overall (phospho)protein abundance [27]. This analysis retained 267 phosphosites that were located on 156 phosphoproteins (Figure 2C). Analogous to the non-normalized phosphoproteome results, the highest number of phosphosites simultaneously detected in two conditions is shared by mock and PSV-P and satRNA samples (104), while the number of phosphosites that were common between the three conditions was lower (68) (Figure 2C). Two-sample comparisons revealed a greater number of down-regulated phosphoproteins than up-regulated phosphoproteins in both PSV-P- and PSV-P and satRNA-treated plants (Table 1, Table S1K–M). Similar to the phosphoproteomic results, normalization confirmed that the addition of satRNA into the virus inoculum led only to the up-regulation of phosphorylation, and no down-regulated phosphosites were found to be statistically significant.

**Figure 7:**
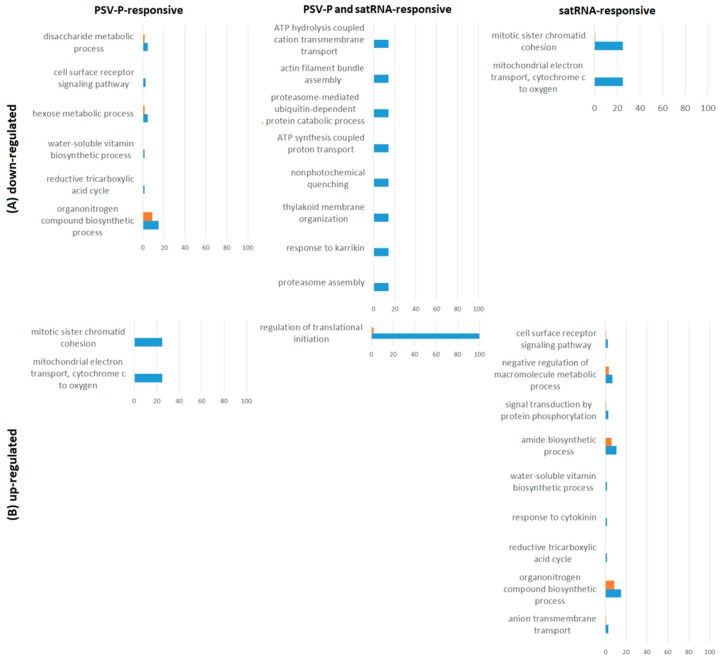
GO enrichment for biological process for phosphoproteins after normalization to protein levels. The distribution of GO terms was analyzed separately for down-regulated (**A**) and up-regulated proteins (**B**) using Blast2GO Basic.

Proteins exclusively present (up- or down-regulated) in one condition (Table S2) were submitted to GO enrichment analysis. Some of the GO terms, for example, carbohydrate metabolic process-related terms (disaccharide and hexose), mitochondrial electron transport, tricarboxylic acid cycle, and signal transduction by protein phosphorylation, matched the phosphoproteome enrichment analysis results without normalization for the corresponding deregulated comparisons. However, some other terms were obtained. Additional terms for PSV-P-responsive proteins with down-regulated phosphopeptides were classified as the organonitrogen compound biosynthetic process and the cell surface receptor signaling pathway (Figure 7), whereas PSV-P and satRNA-responsive proteins with down-regulated phosphopeptides were enriched in the following processes: proteasome-related terms (proteasome assembly and proteasome-mediated ubiquitin-dependent protein catabolic process), thylakoid membrane organization, and nonphotochemical quenching. Meanwhile, up-regulated phosphoproteins were enriched in regulating translational initiation.

The KAAS analysis confirmed the pathways analyzed for the phosphoproteome without normalization to protein levels (Figure 4). However, in the case of PSV-P and satRNA-responsive proteins with down-regulated phosphopeptides, additional pathways regulated by phosphorylation were detected, such as oxidative phosphorylation (ko00190) and those related to proteasome (ko03050).

### 2.5. Evaluation of Correlation between Gene Expression and (Phospho)Protein Level

To assess if gene expression contributed to the observed changes in the (phospho)proteome, 19 genes for proteins that may be involved in pathogenesis or defense processes obtained from both proteomic and phosphoproteomic analyses were selected for analysis. ERG3 (elicitor-responsive protein 3) was found to play a role in plant defense signaling, where *ERG3* transcript levels were greatly enhanced by treatment of rice with a fungal elicitor and a Ca^2+^-ionophore [30]. BIP (binding immunoglobulin protein, heat shock 70 kDa protein) and PR2B (glucan endo-1,3-β-glucosidase) belong to the stress response-associated proteins [3,31]. Another group of proteins that play essential roles in plant-host defense are Argonaute proteins (AGO1B and AGO4). They participate in post-transcriptional gene silencing, a process that is activated in response to the presence of double-stranded RNAs (dsRNAs) in plants [32]. Finally, increased activity of PPC1 (phosphoenolpyruvate carboxylase 1) was shown to correlate with the development of viral infection and reduction of photosynthesis [33].

Our RT-qPCR analysis results showed that the direction of the changes in transcript level does not always correlate with protein level, and protein or transcript level does not always correlate with phosphoprotein level (Table 2). Mostly, for phosphoproteins that were statistically significant, their corresponding proteins were not found to be altered. The differences in the direction of the changes may result from transcription or translation efficiency, post-transcriptional or translational regulation, or mRNA or protein degradation.

### 2.6. Analysis of Protein–Protein Interaction Networks in PSV-P and PSV-P and satRNA-Infected N. benthamiana

**Figure 8:**
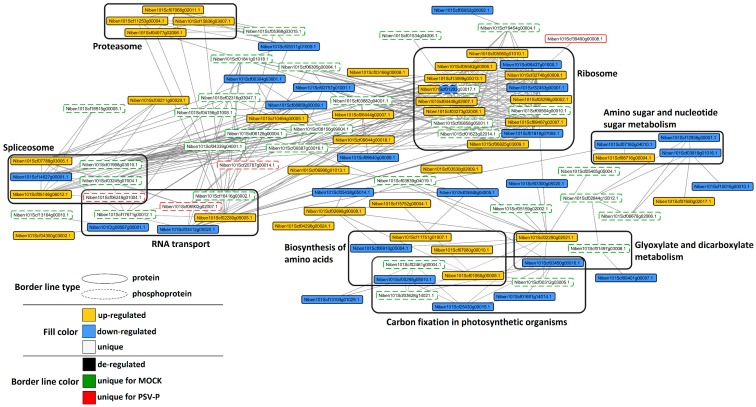
Protein–protein interaction networks of *N. benthamiana* PSV-P-responsive (phospho)proteins, differentially changed in their abundance. Cytoscape was used for visualization with SolGenomics *N. benthamiana* identifiers for (phospho)proteins. Grouping according to KEGG pathways was done for at least three interacting (phospho)proteins.

**Figure 9:**
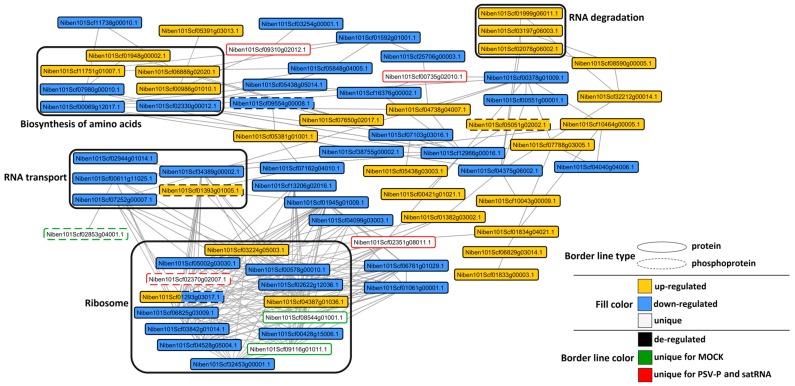
Protein–protein interaction networks of *N. benthamiana* PSV-P and satRNA-responsive (phospho)proteins, differentially changed in their abundance. Cytoscape was used for visualization with SolGenomics *N. benthamiana* identifiers for (phospho)proteins. Grouping according to KEGG pathways was done for at least three interacting (phospho)proteins.

To investigate possible interactions between PSV-P- or PSV-P and satRNA-responsive proteins and phosphorylated proteins normalized to proteome data and to cluster sub networks into several KEGG groups, STRING (search tool for recurring instances of neighbouring genes) analysis was performed. At the early stage of the PSV-P infection process, most interacting (phospho)proteins were involved in ribosome, carbon fixation in photosynthetic organisms, RNA transport, biosynthesis of amino acids, amino sugar and nucleotide sugar metabolism, spliceosome, and proteasome (Figure 8). Whereas in PSV-P and satRNA *N. benthamiana* plants at 5 dpi, the interacting (phospho)proteins were mostly involved in ribosome; biosynthesis of amino acids; RNA transport; and, in contrast to PSV-P-infected plants, RNA degradation (Figure 9).

## 3. Discussion

Our previous studies on PSV-G strain- and PSV-G and satRNA-infected *N. benthamiana* transcriptomes showed that in the PSV-G-infected plants, more of the differentially expressed genes (DEGs) associated with phosphorylation were found to be down-regulated compared with PSV-G and satRNA-infected plants, where the number of up-regulated DEGs associated with this category was considerably higher [34]. This result indicates that phosphorylation may be one of the most significant process connected to satRNA influence on plant defense mechanisms against PSV infection.

### 3.1. PSV-P and PSV-P and satRNA Infections Influenced N. benthamiana Signaling Pathways

Phosphorylation is one of the most important post-translational modifications for cell signaling. The pathogenesis process may lead to disturbances in intra- and inter-celllular communication; therefore, the changes in signaling pathways caused by PSV-P and PSV-P and satRNA were analyzed. In our studies, KAAS analysis indicated an involvement of deregulated proteins and phosphoproteins in PI3K–Akt, MAPK, and mTOR signaling pathways in PSV-P and PSV-P and satRNA-treated plants. A comparison between PSV-P-, PSV-P and satRNA-, and mock-infected plants showed strong down-regulation of protein phosphorylation levels in PSV-P-treated plants, whereas satRNA presence in virus inoculum led to up-regulation in phosphorylation. GO enrichment analysis revealed that only in PSV-P-infected *N. benthamiana*, a decrease in processes connected with signaling and cell communication processes at the proteome level was found. In contrast, at the phosphosphoproteome level, protein autophosphorylation and signal transduction were found. Specifically, down-regulation of auxin-activated signaling pathways at the proteome level was detected in the aforementioned condition. A study on the *Rice dwarf virus* P2 protein showed that auxin signaling plays an active role in antiviral defense that needs to be repressed by the virus in order to attain productive infection [35]. Another term, the G-protein coupled signaling pathway, was also a down-regulated term at the proteome level. Studies concerning G-protein coupled signaling in response to different pathogen elicitors in *N. benthamiana* indicated that G-proteins positively regulated defense-related genes in plant defense signaling [36]. Moreover, among PSV-P-responsive phosphoproteins, the down-regulated term “cell surface receptor signaling pathway” was found, with which receptor-like protein kinases (RLK) are associated. RLKs in plants function as a central processing units, accepting parallel input signals and converting them into appropriate output signals that lead to changes in metabolism, gene expression, or development [37]. RLKs were found to be involved in plant defense [38].

Viruses have developed multiple mechanisms to activate TOR signaling in favor of viral translation. One such strategy resulted in stimulation of the PI3K–AKT pathway upstream of the TOR kinase [39]. In our proteome and phosphoproteome data, the TOR signaling pathway was also affected by PSV-P and PSV-P and satRNA infections. There are different requirements for activation of TOR signaling, as was shown in studies on *Watermelon mosaic virus* (WMV) and *Turnip mosaic virus* (TuMV). Silencing of the *TOR* gene in *Arabidopsis thaliana* severely altered WMV accumulation, while TuMV accumulation was only slightly delayed [40]. Also, MAPK (mitogen-activated protein kinase) signaling pathways, found to be affected in this study (KAAS analysis), are of importance for plant defense. MAPK cascades were found to be involved in signaling pathways by which signals generated by biotic/abiotic stimuli are transduced into intracellular responses [41]. It was shown that some of the components of the MAPK cascade function as positive regulators of PVX-PVY (*Potato virus X*-*Potato virus Y*)-induced cell death. Nevertheless, ameliorated cell death induced by PVX-PVY in the MAPK(K)-silenced plants did not facilitate virus accumulation in systemically infected leaves [42].

In case of the PSV-P and satRNA-treated plants, where lower levels of viral RNAs were observed, proteins involved in jasmonic (JA) and salicylic acid (SA) pathways were found to be up-regulated in the phosphoproteome data. Among proteins detected in our study, the regulator of nonsense transcripts 2 (UPF2, Niben101Scf01035g06018.1), required for nonsense-mediated decay, was also previously found to be involved in stress response in *Arabidopsis* exposed to wounding and infection by *Pseudomonas syringae* [43]. Additionally, the nonsense-mediated decay pathway is a highly conserved mRNA surveillance pathway able to detect and degrade some viral RNAs [44,45]. Nonsense-mediated decay may be meaningful for the PSV-P and satRNA pathogenesis in *N. benthamiana*, as our STRING analysis of the (phospho)proteome also marked few interacting proteins involved in RNA degradation. Integration of all of these pathways might have led to the attenuation of multiplication of PSV-P and satRNA.

An additional group of differentially regulated proteins in our proteomic analysis is 14-3-3 proteins, a eukaryotic-specific protein family binding to phosphopeptides. They act as sensors for phosphorylated motifs, but also undergo phosphorylation. They are involved in primary metabolism, signal transduction, cell cycle control, apoptosis, and stress responses, among others [46]. They are also indicated to play a role in plant immune responses. These 14-3-3 proteins were identified as putative key regulators of the response to *Tomato yellow leaf curl virus* infecting tomato plants [47].

### 3.2. PSV-P and PSV-P and satRNA Manipulate Primary Metabolism

During PSV-P-plant interactions, photosynthesis, carbon fixation, and other processes related to chloroplasts were strongly down-regulated, mostly at the proteome level. However, in the presence of satRNA, more proteins with decreased levels were found, implying that this small particle presence contributes to changes in the plant metabolism during viral infection. Photosynthesis or other processes associated with chloroplasts were reported to be affected by numerous viruses including *Cucumber mosaic virus* (CMV), PSV, or *Tobacco mosaic virus* (TMV), suggesting a common phenomenon [11,12,48,49]. Usually, viruses decrease photosynthetic activity, but satRNA presence strengthens the down-regulating effect, which was shown in PSV-P and satRNA-infected *N. benthamiana* proteomic studies [12].

Our results showed that amino acid biosynthesis was also affected by PSV-P and PSV-P and satRNA infections (both by up- and down-regulated proteins, to a lesser extent at the phosphoproteome level). However, the satRNA presence in the virus inoculum caused stronger down-regulation. Histidine biosynthesis was down-regulated at the proteomic level in PSV-P and satRNA-infected plants. Changes, up- and down-regulation, in processes associated with amino acid metabolism were reported in multiple studies. Infections with *Squash mosaic virus* or *Tobacco rattle virus* caused an increase in amino acid biosynthesis or content [50,51]. However, in some other studies, the levels of certain enzymes connected with amino acid metabolism were observed to be decreased, for example, upon *Papaya meleira virus* (PMeV) infection [52].

The levels of proteins associated with translation and ribosomes were strongly influenced by PSV presence in this and our previous studies [34]. At the proteomic level, satRNA caused a decrease in proteins connected with translation-associated terms, for example, regulation of elongation and ribosomal large subunit assembly, while at the phosphoproteome level, proteins connected with translation initiation were up-regulated in phosphorylation. Translation initiation and elongation factors were differentially regulated during infection with many viruses, for example, PMeV and *Sugarcane mosaic virus* (SCMV) [13,52]. Additionally, levels of diverse ribosomal proteins varied during infection with *Beet necrotic yellow vein virus* (BNYVV) or SCMV [53,54]. Among many ribosomal proteins, 40S ribosomal protein S6 (RPS6, Niben101Scf01293g03017.1), which was found to be the strongly up-regulated protein and down-regulated in phosphorylation under PSV-P or PSV-P and satRNA infection in our analysis, has attracted significant attention. The protein level and phosphorylation status of RPS6 were examined in *N. benthamiana* plants infected with CMV, TMV, TuMV, *Turnip crinkle virus*, and *Potato virus A*. All of the viruses were able to induce the accumulation of the protein. However, no obvious changes in phosphorylation level of RPS6 after infection with these viruses were observed. Possibly because the experimental system that was used might have lacked sufficient sensitivity or resolution to detect changes in phosphorylation [26], as RPS6 in most cases of human or animal viral infections was strongly dephosphorylated within hours after infection [55]. Therefore, the lower accumulation level of PSV-P and satRNA in *N. benthamiana* plants may be associated with down-regulation of biosynthesis of amino acids, as well as with ribosomes, causing down-regulation of translation activity of viral proteins.

### 3.3. N. benthamiana Plants Respond with Changes in the Level and Phosphorylation Status of Stress Response-Associated Proteins

An important part of the plant’s reaction to biotic and abiotic factors is a set of stress-responsive proteins. Reactive oxygen species (ROS) and ROS-scavenging enzymes (enzymes that control ROS) belong to this group of proteins. At the proteomic level, in response to satRNA presence, the levels of proteins such as superoxide dismutases (SOD), peroxidases, and thioredoxins were up-regulated and associated with oxidative stress and cellular oxidant detoxification terms. Likewise, SOD and peroxidase levels were found to be present at higher levels in plants infected with pepper mild mottle virus (PMMoV) or BNYVV [53,56].

Another group of stress-responsive proteins is chaperones and related proteins, which are frequently identified in plant-virus proteomic studies. The chaperone-mediated protein complex was the GO enriched term among down-regulated proteins in PSV-P-treated plants, while among satRNA-responsive proteins, a heat shock protein was detected in the proteome analysis. Plants infected by various plant viruses express cytosolic Hsp70 proteins at elevated levels, indicating that Hsp70 could play an important role during viral infections. However, proteome studies of PSV-P- and PSV-P and satRNA-infected *N. benthamiana* after symptom occurrence revealed different forms of Hsp70 with one elevated and the other less abundant in response to satRNA [12,31,57].

Some proteins induced by pathogen attack have been classified as pathogenesis-related (PR) proteins. There are many classes of PR proteins and a few have been identified in our proteomic analysis in PSV-P and satRNA-treated plants. High up-regulation and GO enrichment of proteins involved in negative regulation of endopeptidase activity and cell wall macromolecule catabolic process were detected. A group of proteinase inhibitors (PR-6), connected with the first term, are differentially regulated during infection with CMV or PMeV [52,58]. Whereas to the latter term, a group of chitinases (PR-3, 4, 8, and 11) was assigned in our study. There were cases when proteins with chitinase activity were up-regulated during PMMoV or RBDSV infections, but also they tended to be down-regulated at the protein level [56,59]. Another PR protein, glucan endo-1,3-β-glucosidase B (PR-2) was found to be less abundant among satRNA-responsive proteins, while in most other studies, PR-2 proteins were more abundant [60,61]. The difference between PR-2 levels in plants infected with PSV-P and PSV-P and satRNA might result from lower accumulation of viral genomic strands during PSV-P and satRNA infection, and this was supported by the enrichment of down-regulated ‘defense response to virus’ GO term.

### 3.4. PSV-P and PSV-P and satRNA Infection Results in Changes in RNA Processing

Plant viruses utilize the host replication machinery for their efficient replication. Viruses have developed means to modulate host cellular processes to their own advantages; however, hosts also exploited antiviral defense mechanisms [62]. Our study revealed changes in several processes connected with RNA synthesis, transport, and turnover (mRNA surveillance pathway, RNA degradation, and spliceosome), which demonstrates that PSV-P, a (+)ssRNA virus, affected *N. benthamiana* RNA-based processes. Most of these processes were discussed above, except the spliceosome. The spliceosome is a protein complex participating in splicing. Alternative splicing was found to play an important role in plant antiviral responses concerning a few individual resistance genes [63,64]. Splicing mutants of the translation initiation factor eIF4E in tomato were shown to be immune against *Potato* virus Y and *Pepper mottle virus* [65].

In conclusion, we provide the first global insight into the early signaling mechanisms associated with PSV pathogenesis, which will be valuable for further studies.

## 4. Materials and Methods

### 4.1. Plant and Virus Materials

*N. benthamiana* plants were grown in controlled conditions in separate greenhouse chambers with 14 h light/10 h dark in 21 °C day/16 °C night cycles. Six four-leaf-stage seedlings per condition were carborundum-dusted and rub-inoculated with 10 µg of PSV-P infectious clones, alone or in combination with 1 µg of satRNA-P infectious clone (obtained as previously described [12]), in 0.05 M phosphate buffer, pH 7.5. Mock-inoculated (0.05 M phosphate buffer, pH 7.5) plants were used as controls. Plant material (all leaves including the inoculated ones) was harvested at five days post inoculation (dpi) and subjected to further (phospho)proteomic analyses.

### 4.2. RT-qPCR for Viral Genomic RNAs and N. benthamiana Transcripts Analyses

Samples for RT-qPCR for viral genomic RNAs and *N. benthamiana* transcripts analyses and reactions setups were prepared as described in Protocol S1.

Detection of PSV-P and satRNA-P was done by means of RT-qPCR as described in Protocol S1. For comparison of accumulation of PSV-P genomic RNAs and satRNA-P, the primer pairs for amplification of RNA 1 (PSVq1), RNA 2 (PSVq2a and PSVq2b), RNA 3 (PSVq3a and PSVqCP), and satRNA (PARNA) listed in Table S3 were used. The levels of viral RNAs were normalized to the levels of EF1α and β-actin mRNAs by amplification with primers NbEF1aF/R and NbActA/2, respectively (Table S3). For each set of primers, the standard curve was obtained from a series of 10-fold dilutions of the respective templates. Plasmids containing PSV-P infectious clones and PCR products of EF1α and β-actin were used to generate standard curves for viral and satellite RNAs detection primers, and EF1α and β-actin primers, respectively [12]. Relative RNA accumulation was calculated by dividing the number of copies of viral RNA or satRNA by the β-actin and EF1α copy numbers. Based on the PSV-P genomic strands accumulation levels, out of six infected plants of similar size and virus accumulation level, three of them were used for subsequent analysis.

Validation of gene expression for chosen transcripts from (phospho)proteome data was done on the pooled samples of the inoculated leaves and the ones above, which were collected at 5 dpi. Plant material for the analysis was prepared as for the assessment of virus accumulation level. cDNA preparation and RT-qPCR reactions were performed as described above. Primers of analyzed genes of chosen transcripts (Table S4) were designed using Primer3 software [66]. Tested genes were normalized to *EF1α* and *β-actin* genes expressions with NbEF1aF/R and NbActA/2 primers (Table S3). The reaction results were analyzed using the Relative Expression Software Tool V.2.0.13 (Qiagen, Hilden, Germany).

### 4.3. Protein Extraction

Protein extraction was performed as described previously [67] with some modifications. Briefly, 1 g of finely ground plant material was suspended in homogenization buffer containing 50 mM Tris-HCl buffer (pH 8), 30% sucrose, 5 mM EDTA, and 1 mM DTT in Milli-Q water and appropriate amounts of the cOmplete^TM^ Protease Inhibitor mixture (Roche, Basel, Switzerland) and the PhosSTOP Phosphatase Inhibitor mixture (Roche). After sonication and removal of cell debris by centrifugation at 4 °C for 15 min at 2500 *g*, a methanol/chloroform precipitation was carried out on the supernatants described previously. Protein pellets were dissolved in 8 M urea. Carbamidomethylation of cysteines was carried out in the presence of 15 mM tris(carboxyethyl)phosphine (TCEP, Pierce) and 30 mM iodoacetamide (Sigma-Aldrich, St. Louis, MO, USA). Samples were diluted to 1 M urea using 50 mM TEAB. Two milligrams of protein material was digested with 10 µg of MS Grade Endoproteinase-Lys-C (Wako Chemicals GmbH, Neuss, Germany) for 4 h, followed by an overnight digestion with 30 µg of trypsin (Promega, Madison, WI, USA) at 37 °C. The digest was acidified to pH ≤ 3 with trifluoroacetic acid (TFA) and desalted using SampliQ C18 SPE cartridges (Santa Clara, CA, Agilent Technologies), according to the manufacturer’s guidelines. The desalted peptides were fully dried in a vacuum centrifuge and then dissolved in 500 µL of loading solvent (80% (*v*/*v*) acetonitrile, 6% (*v*/*v*) TFA), from which 100 µL was kept for proteome analysis, while 400 µL was processed further with phospho-enrichment using MagReSyn^®^ Ti-IMAC (ReSyn Biosciences, Edenvale, Gauteng, South Africa) as described previously [68]. Samples were vacuum dried and re-dissolved in 30 µL 2% (*v*/*v*) acetonitrile and 0.1% (*v*/*v*) TFA prior to LC-MS/MS analysis.

### 4.4. Mass Spectrometry

LC-MS/MS analysis was performed as previously described [27]. For details, see Supporting Information (Protocol S2).

### 4.5. (Phospho)Proteomic Data Analysis

The database search (using the *N. benthamiana* proteome downloaded from SolGenomics database containing 57,140 protein entries) [69] and data analysis were performed using MaxQuant v.1.5.4.1 [70] and Perseus 1.5.6.0 (available in the MaxQuant package), respectively, as described previously [27]. For phosphoproteome data, only phosphosites with localization probabilities larger than 0.75 were retained for further analysis. Two-sample tests between conditions were performed with a cut-off *p*-value of 0.05. All MS proteomics data have been deposited to the ProteomeXchange Consortium (http://www.ebi.ac.uk/pride/archive/) via the PRIDE partner repository [71,72] with the dataset identifier PXD010675. Normalization of phosphoproteome data was done as described previously [27].

### 4.6. Motif-X Analysis

The Motif-X algorithm was used to extract statistically overrepresented amino acid motifs surrounding the phosphosites identified in the two-sample tests (*p*-value < 0.05) [73]. The sequence window was limited to 13 amino acids, and foreground peptides were pre-aligned with the phosphosite in the center. All phosphoproteins identified in the experiment were used as the background database. The occurrence threshold was set at the minimum of 20 peptides, and the *p*-value threshold was set at <10^−6^.

### 4.7. Functional Annotation of Differentially Expressed Genes and Pathway Analysis

Proteins and phosphoproteins, which differed between non-infected plants and those infected with PSV-P or PSV-P and satRNA-P, were pinpointed as statistically different (*p*-value < 0.05) and were uploaded to the Blast2GO Pro software [74] as queries in blastx searches against the NCBI nucleotide collection (nr) (https://blast.ncbi.nlm.nih.gov/Blast.cgi?PROGRAM=blastn&PAGE_TYPE=BlastSearch&LINK_LOC=blasthome) and InterPro databases [75]. Mapping, annotation, and classification into functional categories were carried out using default settings in Blast2GO Pro. Annex-based gene ontology (GO) augmentation enhanced annotations. Thereafter, KAAS, the KEGG Automatic Annotation Server [28], was used to classify particular (phospho)proteins into appropriate processes and pathways by analyzing obtained results to which KEGG enzyme codes were assigned against data obtained from the KEGG pathway database.

Additionally, GO enrichment was performed using Blast2GO Basic with default settings. Proteins; phosphoproteins and phosphoproteins normalized to protein levels, the levels of which were significantly changed (*p*-value < 0.05); and unique (phospho)proteins that were found exclusively as PSV-, PSVsat-, and sat-responsive were used for further analysis. Protein sequences of all identified proteins or phosphoproteins were loaded in Blast2GO software and blasted against the NCBI nr protein sequence database of green plants (Viridiplantae) with default settings. Afterwards, the results were examined for GO annotation followed by Fisher’s exact test (*p* < 0.05) to extract enriched GO terms.

### 4.8. STRING Analysis of Protein–Protein Interaction Networks

Protein–protein interaction networks were constructed based on STRING analysis (https://string-db.org) [76]. Sequences of differentially regulated and unique PSV-P- and PSV-P and satRNA-responsive proteins and phosphoproteins (normalized to proteins levels), for which the level significantly changed in our data sets, were used as input. *Solanum tuberosum* L. was set as reference organism. The minimum required interaction score was set as high confidence (>0.700) and the active interaction sources were chosen as follows: textmining, experiments, databases, co-expression, gene fusion, and co-occurrence. The results were visualized using Cytoscape (version 3.6.0) [77] with *S. tuberosum* ID numbers converted to *N. benthamiana* ID numbers from SolGenomics database [69].

## Figures and Tables

**Table 1:** Differentially regulated (phospho)proteins.

Treatment	Proteome		Phosphoproteome		Phosphoproteome after Normalization	
	Up	Down	Up	Down	Up	Down
PSV-P-responsive	111	54	4	87	2	20
PSV-P and satRNA-responsive	66	87	37	34	2	15
satRNA-responsive	56	218	84	4	19	-

*N. benthamiana* infected with peanut stunt virus (PSV)-P and PSV-P and satellite RNA (satRNA). PSV-P-responsive, PSV-P and satRNA-responsive, and satRNA-responsive (phospho)proteins extracted by comparison of (phospho)proteomes of PSV-P with MOCK, PSV-P and satRNA with MOCK, and PSV-P and satRNA with PSV-P, respectively.

**Table 2:** Validation of the proteomic (light grey) and phosphoproteomic (grey) data by gene expression levels analysis (quantitative reverse transcriptase real-time polymerase chain reaction—RT-qPCR).

Validation				Proteomics		Phosphoproteomics	
**Gene**	Expression	P(H1)	Result	Protein Level	Result	Phosphorylation Level	Result
**PSV-P-responsive**							
BIP	1.131	0.085		1.384	UP		
ERG3	0.997	0.941		2.970	UP		
MCA	0.949	0.133		1.552	UP		
BSL3-like	1.108	0.015	UP	1.040		2.461	UP
FBP2like	1.151	0.117		0.788		0.062	DOWN
PMI1	1.284	0.005	UP	1.213		0.313	DOWN
PPC1	1.249	0.012	UP	0.975		0.152	DOWN
RS6	0.833	0.010	DOWN	3.340	UP	0.275	DOWN
TPR-like1320	1.122	0.106		1.283	UP	0.250	DOWN
TSJT1	0.722	0	DOWN	1.073		0.282	DOWN
**PSV-P and satRNA-responsive**							
BIP	1.469	0	UP	1.279	UP		
GRP2	1.221	0	UP	1.204	UP		
PR2B	0.580	0.018	DOWN	0.300	DOWN		
AGO1B	1.166	0.004	UP	1.268		0.556	DOWN
EIF5	1.200	0	UP	1.300		2.069	UP
ECT5	1.109	0.012	UP	1.053		0.507	DOWN
RPN10	0.956	0.118		1.330		0.397	DOWN
RS6	0.903	0.062		2.848	UP	0.312	DOWN
TPR-like1320	1.122	0.130		1.223		0.616	DOWN
**satRNA-responsive**							
AGO4	1.169	0.002	UP	0.757	DOWN		
ERG3	1.446	0	UP	0,359	DOWN		
GRP2	1.162	0.002	UP	1.299	UP		
PR2B	1.202	0.345		0.201	DOWN		
PSB	1.207	0	UP	0.839	DOWN		
FBP2like	0.914	0.309		1.369		12.957	UP
PGM1	0.980	0.682		0.850		3.042	UP
PMI1	0.849	0.012	DOWN	0.771		3.285	UP
PPC1	0.897	0.222		0.835		5.959	UP
TSJT1	1.148	0.001	UP	0.813		3.957	UP

The results UP or DOWN indicate the direction of statistically important changes. Expression as well as protein and phosphorylation levels values represent fold changes.

## Supplementary Materials

Supplementary materials can be found at http://www.mdpi.com/1422-0067/19/10/3223/s1. Figure S1: Accumulation level analysis of PSV-P RNAs and satRNA in *N. benthamiana* plants infected with PSV-P and PSV-P and satRNA; Table S1: Lists of reproducibly quantified (phospho)proteins, differentially regulated PSV-P-, PSV-P and satRNA-, and satRNA-responsive proteins, and uniquely identified (phospho)proteins between conditions; Table S2: (Phospho)proteins found exclusively in one of the conditions during pairwise comparisons; Table S3: Primers used for virus and satellite detection/accumulation measurements by RT-qPCR; Table S4: Primers used for validation of chosen transcripts from (phospho)proteomic results; Protocol S1: Samples preparation and RT-qPCR procedures; Protocol S2: Mass spectrometry procedure.
