# Supplementary material for: Peanut Stunt Virus and Its Satellite RNA Trigger Changes in Phosphorylation in N. benthamiana Infected Plants at the Early Stage of the Infection
Source: Int J Mol Sci. 2018 Oct 18;19(10):3223. doi: 10.3390/ijms19103223 (PMC6214028; doi:10.3390/ijms19103223)
Supplement: Supplementary file 1 [file ijms-19-03223-s001.zip › ijms-366166-SI.pdf]

Peanut Stunt Virus and its Satellite RNA Trigger Changes in Phosphorylation in *Nicotiana benthamiana* Infected Plants at the Early Stage of the Infection

Supplementary Material

Barbara Wrzesińska[1], Lam Dai Vu[2,3,4,5], Kris Gevaert[4,5], Ive De Smet[2,3], Aleksandra Obrępalska-Stęplowska[1,]*

[1]Institute of Plant Protection – National Research Institute, Department of Entomology, Animal Pests and Biotechnology, Władysława Węgorka 20, 60-318 Poznań, Poland
[2]Ghent University, Department of Plant Biotechnology and Bioinformatics, Technologiepark 927, 9052 Ghent, Belgium
[3]VIB Center for Plant Systems Biology, Technologiepark 927, 9052 Ghent, Belgium
[4]Department of Biomolecular Medicine, Ghent University, B-9000 Ghent, Belgium
[5]VIB Center for Medical Biotechnology, B-9000 Ghent, Belgium

*Correspondence :
Dr. Aleksandra Obrępalska-Stęplowska
olaob@o2.pl or ao.steplowska@iorpib.poznan.pl

Table of Contents

Supplementary Figure S1. Accumulation level analysis of PSV-P RNAs and satRNA in *N. benthamiana* plants infected with PSV-P and PSV-P+satRNA 3

Supplementary Protocol S1. Samples preparation and RT-qPCR procedures 4

Supplementary Protocol S2. Mass spectrometry 5

Supplementary Table S1. Primers used for virus and satellite detection/accumulation measurements by RT-qPCR 6

Supplementary Table S2. Primers used for validation of chosen transcripts from (phospho)proteomic results 7

Supplementary Table S4. (Phospho)proteins found exclusively in one of the conditions during pairwise comparisons 11

References 12

Supplementary Figure S1. Accumulation level analysis of PSV-P RNAs and satRNA in *N. benthamiana* plants infected with PSV-P and PSV-P+satRNA. *N. benthamiana* plants were infected with biologically infectious transcripts of PSV-P (blue boxes) or PSV-P+satRNA (orange boxes). The RT-qPCR analysis was done to show changes in the levels of PSV-P genomic strands (RNA 1 – q1, RNA 2 – q2a and q2b, RNA 3 – q3a and qCP, and satRNA) between plants infected with virus and satRNA, and virus alone. The error bars represent standard errors; * - statistically significant results.

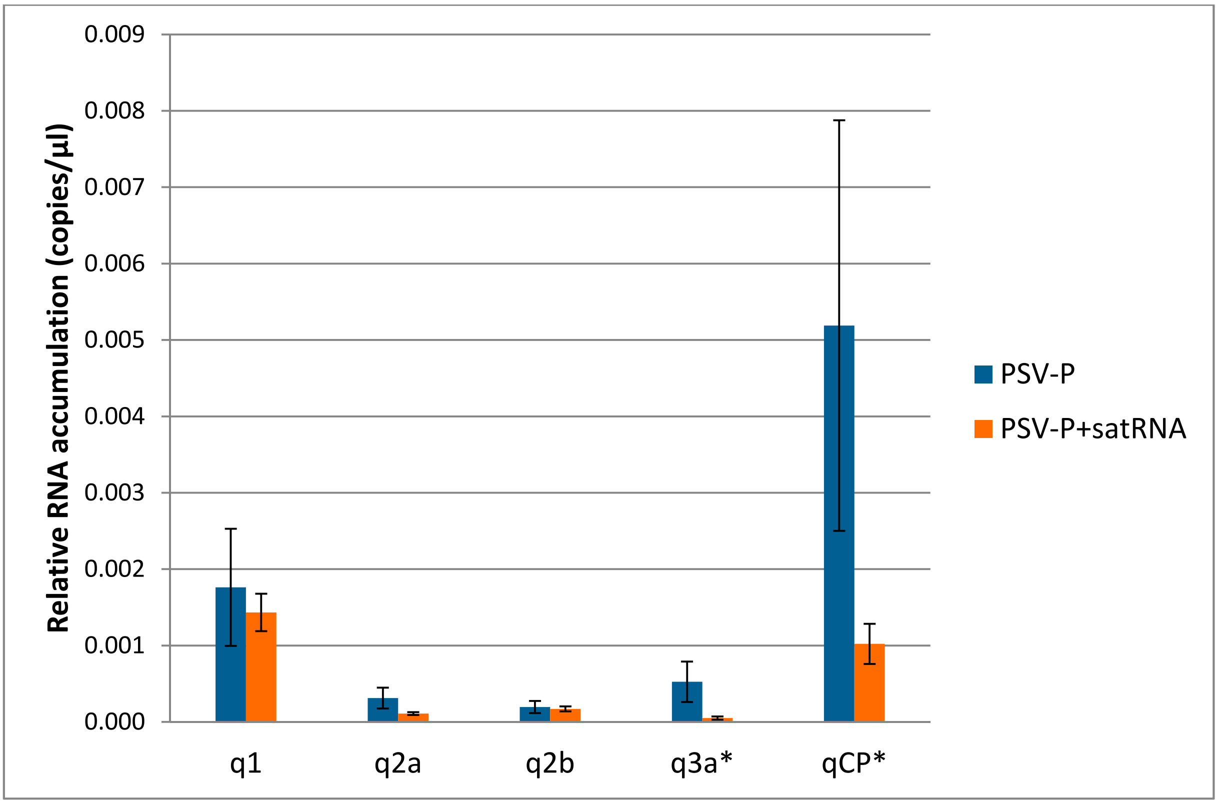

Supplementary Protocol S1. Samples preparation and RT-qPCR procedures.

Total RNA was extracted from the harvested plants using Tri Reagent solution (Thermo Fisher Scientific, Waltham, MA, USA) followed by genomic DNA digestion as previously described [1]. One μg of purified RNA was reverse transcribed using RevertAid Reverse Transcriptase (Thermo Fisher Scientific) with a random-sequence primer (5'-NNNNNN-3', Thermo Fisher Scientific). The resulted cDNA samples (20 μL) were diluted with 20 μL DNase-free water and used for RT-qPCR. The reactions were completed in a LightCycler 480 (Roche, Basel, Switzerland). The reaction was conducted in a 10-μL solution using iTaq™ Universal SYBR® Green Supermix (BioRad, Hercules, CA, USA) with 0.5 μM forward and reverse primers, and 1 μl of diluted cDNA. The reaction profile consisted of an initial denaturation step at 95 °C for 3 min, followed by 40 cycles of 95 °C for 20 s, an annealing step for 20 s (temperatures listed in supplemental tables), and 72 °C for 20 s.

Supplementary Protocol S2. Mass spectrometry procedure

Each sample was analyzed via LC-MS/MS on an Ultimate 3000 RSLC nano LC (Thermo Fisher Scientific) in-line connected to a Q Exactive mass spectrometer (Thermo Fisher Scientific). The peptides were first loaded on a trapping column (made in-house, 100 µm internal diameter (I.D.) × 20 mm, 5 µm beads C18 Reprosil-HD (Dr. Maisch, Ammerbuch-Entringen, Germany). After flushing the trapping column, peptides were loaded in solvent A (0.1% formic acid in water) on a reverse-phase column (made in-house, 75 µm I.D. x 250 mm, 1.9 µm Reprosil-Pur-basic-C18-HD beads, Dr. Maisch, packed in the needle) and eluted by an increase in solvent B (0.1% formic acid in acetonitrile) in a linear gradient from 2% solvent B to 55% solvent B in 120 minutes, followed by a 5-min washing step with 99% solvent B, all at a constant flow rate of 300 nl/min. The mass spectrometer was operated in data-dependent, positive ionization mode, automatically switching between MS and MS/MS acquisition for the 5 most abundant peaks in a given MS spectrum. The source voltage was set at 4.1 kV and the capillary temperature at 275°C. One MS1 scan (m/z 400−2,000, AGC target 3 × 106 ions, maximum ion injection time 80 ms), acquired at a resolution of 70,000 (at 200 m/z), was followed by up to 5 tandem MS scans (resolution 17,500 at 200 m/z) of the most intense ions fulfilling predefined selection criteria (AGC target 5 × 104 ions, maximum ion injection time 80 ms, isolation window 2 Da, fixed first mass 140 m/z, spectrum data type: centroid, under-fill ratio 2%, intensity threshold 1.3xE4, exclusion of unassigned, 1, 5-8, >8 positively charged precursors, peptide match preferred, exclude isotopes on, dynamic exclusion time 12 s). The HCD collision energy was set to 25% Normalized Collision Energy and the polydimethylcyclosiloxane background ion at 445.120025 Da was used for internal calibration (lock mass).

Supplementary Table S1. Primers used for virus and satellite detection/accumulation measurements by RT-qPCR.

Primer name	Primer sequence (5'-3')	Annealing temp. [□C]	Function
PSVq1	F: CTTCTGCCCTCGTTGATAAAG<br>R: CATACCGATTTCGAATCACTT	57	Detection of PSV 1a protein ORF in RT-qPCR [2]
PSVq2a	F: CTTCTAGGTATCCCCGTAAG<br>R: CAAGCACATTGATACCCTATC	60	Detection of PSV 2a protein ORF in RT-qPCR [2]
PSVq2b	F: CTCMTATCCTCCCAGCTAYAC<br>R: GAATAACTRCCCTCACACCAC	53	Detection of PSV 2b protein ORF in RT-qPCR [2]
PSVq3a	F: CTAGTCGGACTTTAACACAAC<br>R: ACGCTCATATATCCCTTAGAC	56	Detection of PSV 3a protein ORF in RT-qPCR [2]
PSVqCP	F: ACACATACACTTCGTTGGATG<br>R: CCTCWTCTTCGGAAATTCAG	55	Detection of PSV coat protein ORF in RT-qPCR [2]
PARNA	1: GGGAGGGCGGGCGTTCGTAGTG<br>2: GCCGTGGCCTTTCGTGGTC	60	satRNA detection in RT-qPCR [2]
NbAct	A: GTGAAGGAGAAGTTGGCTTAC<br>2: CTTCTGGGCAGCGGAATCTC	60	β actin amplification in RT-qPCR [3]
NbEF1a	F: CACCATTGATATTGCCTTGTG<br>R: GTTCTTGATAAAGTCCCTGTG	53	elongation factor 1α amplification in RT-qPCR [4]

Supplementary Table S2. Primers used for validation of chosen transcripts from (phospho)proteomic results (* - primers for 40S ribosomal protein S6 and tetratricopeptide repeat (TPR)-like superfamily protein genes, which hits were found to be statistically significant in both proteomic and phosphoproteomic analysis).

Primer name	Primer sequence (5’-3’)	Annealing temp. [□C]	Amplicon length [bp]	Gene annotation with SolGenomics accession number
Proteome validation				
NbAGO4	F: TGAAGAAAAAGGCGGCTCTA<br>R: GTGTCCATCCACATTGGTCA	61	119	protein argonaute 4 (Niben101Scf05519g01007.1)
NbBIP	F: GCTGAAGACAAAGCCTCTGG<br>R: TCCTCCTCTGCAAACTCCTC	61	119	heat shock-related 70 kDa protein 2 (Niben101Scf03115g02008.1)
NbERG3	F: GGAAGGGTTGTGAACCTGAA<br>R: GAAGTCGTCTTCGCCTACAGA	61	113	elicitor-responsive protein 3 (Niben101Scf09044g01005.1)
NbGRP2	F: ATTCGGTACATACGGCGAAG<br>R: AGCATCCCTCATGCATTTCT	56	115	glycine-rich RNA-binding protein 2 (Niben101Scf03214g00006.1)
NbMCA	F:	57	120	metacaspase-4 (Niben101Scf01376g04029.1)

	CAAATCCTTGCCTCTTTCCA			
	R: GGACTAGCATCTTCGCCAAA			
NbPR2B	F: CCCAATTCAGATGTGAAGCA	56	124	glucan endo-1,3-beta-glucosidase B (Niben101Scf01934g02004.1)
	R: TGATTTCATTCCCAACAGCA			
NbPSB	F: CTTCTTGGTGCAAGTGGTGA	57	106	proteasome subunit beta (Niben101Scf15836g03007.1)
	R: GACCCAAAGAGTTCCCATCA			
Phosphoproteome validation				
NbAGO1B	F: AGACAACCACTGGGTGAAGG	60	152	protein argonaute 1B (Niben101Scf05146g06007.1)
	R:TTCAGAAGCTGGCTCACAAA			
NbBSL3like	F: GATGGATGGCTTTGAACGAT	60	150	serine/threonine protein phosphatase family protein (Niben101Scf04699g00014.1)
	R: GGTGGCAATGGGTGAATAAG			
NbECT5	F: CCCGTGGACTCTGGAAGAT	60	152	evolutionarily conserved C-terminal region 5 (Niben101Scf08176g00008.1)

	A<br>R: GAATAATGCCTGGCTGAGGA			
NbEIF5	F: AGGAAGATGGTTCGCAGCTA<br>R: TCCAGATTGGGGAGAGTTTG	60	192	eukaryotic translation initiation factor 5 (Niben101Scf01393g01005.1)
NbFBP2like	F: CCTAAAACAATGGCCGAAGA<br>R: GAGCACCATCAGGAGGAGAG	60	154	polyribonucleotide nucleotidyltransferase (Niben101Scf00394g03001.1)
NbPGM1	F: AAAGGTGCTACGCTTGTGGT<br>R: ACAGCTGATACGGCAGGAGT	60	149	phosphoglucomutase-1 (Niben101Scf01697g23018.1)
NbPMI1	F: CTCGCTCACATTGGTAAGCA<br>R: TCTGGATGGCATGGTTTGTA	60	156	plastid movement impaired1 (Niben101Scf03738g00006.1)
NbPPC1	F: AGCGTGGCAGCTGTATAAGG	63	151	phosphoenolpyruvate carboxylase 1 (Niben101Scf25430g00015.1)

	R: TGTATCGGGTGGTTGAGACA			
NbRPN10	F: CGAGTTTCAATGGAGGAGGA	60	152	26S proteasome non-ATPase regulatory subunit 4 homolog (Niben101Scf06856g00007.1)
	R: GCCTTGTTTTCAGGTTCAGG			
NbRS6*	F: ATCGACGACGACCAGAAACT	63	147	40S ribosomal protein S6 (Niben101Scf01293g03017.1)
	R: TCCCTGCTTCATTGGAAAAC			
NbTPR-like1320*	F: GGACAAAACCGTTCATTTGG	60	149	tetratricopeptide repeat (TPR)-like superfamily protein (Niben101Scf02283g00007.1)
	R: GCCTTCGTCTTCGTCCATAG			
NbTSJT1	F: TCCGAACAATGAGACAGCAG	63	151	aluminium induced protein with YGL and LRDR motifs (Niben101Scf10940g04023.1)
	R: CCTGGGAAGAAGAGGGTTTT			

Supplementary Table S4. (Phospho)proteins found exclusively in one of the conditions during pairwise comparisons. PSV-P-responsive, PSV-P+satRNA-responsive, and satRNA-responsive (phospho)proteins extracted by comparison of (phospho)proteomes of PSV-P with MOCK, PSV-P+satRNA with MOCK, and PSV-P+satRNA with PSV-P, respectively.

Treatment	Proteome		Phosphoproteome		Phosphoproteome after normalization	
	UP	DOWN	UP	DOWN	UP	DOWN
**PSV-P-responsive**	68	32	4	205	5	161
**PSV-P+satRNA-responsive**	33	28	15	10	1	7
**satRNA-responsive**	40	168	203	7	165	5

References

1. Wielkopolan, B.; Krawczyk, K.; Obrępalska-Stęplowska, A. Gene expression of serine and cysteine proteinase inhibitors during cereal leaf beetle larvae feeding on wheat: the role of insect-associated microorganisms. *Arthropod-Plant Interactions* **2018**, 1-12.
2. Obrępalska-Stęplowska, A.; Renaut, J.; Planchon, S.; Przybylska, A.; Wieczorek, P.; Barylski, J.; Palukaitis, P. Effect of temperature on the pathogenesis, accumulation of viral and satellite RNAs and on plant proteome in peanut stunt virus and satellite RNA-infected plants. *Frontiers in Plant Science* **2015**, *6*, doi:10.3389/fpls.2015.00903.
3. Obrępalska-Stęplowska, A.; Wieczorek, P.; Budziszewska, M.; Jeszke, A.; Renaut, J. How can plant virus satellite RNAs alter the effects of plant virus infection? A study of the changes in the *Nicotiana benthamiana* proteome after infection by *Peanut stunt virus* in the presence or absence of its satellite RNA. *Proteomics* **2013**, *13*, 2162-2175.
4. Obrępalska-Stęplowska, A.; Zmienko, A.; Wrzesińska, B.; Goralski, M.; Figlerowicz, M.; Zyprych-Walczak, J.; Siatkowski, I.; Pospieszny, H. The defense response of *Nicotiana benthamiana* to peanut stunt virus infection in the presence of symptom exacerbating satellite RNA. *Viruses* **2018**, *10*, 449.
